# NMR-Based Metabolomic Study on *Phaseolus vulgaris* Flour Fermented by Lactic Acid Bacteria and Yeasts

**DOI:** 10.3390/molecules28124864

**Published:** 2023-06-20

**Authors:** Giuseppina Tatulli, Laura Ruth Cagliani, Francesca Sparvoli, Milena Brasca, Roberto Consonni

**Affiliations:** 1 National Research Council, Institute of Sciences of Food Production (ISPA), Via Celoria 2, 20133 Milan, Italy; giuseppina.tatulli@ispa.cnr.it (G.T.); milena.brasca@ispa.cnr.it (M.B.); 2 National Research Council, Institute of Chemical Sciences and Technologies “G. Natta” (SCITEC), Via Corti 12, 20133 Milan, Italy; roberto.consonni@scitec.cnr.it; 3 National Research Council, Institute of Agricultural Biology and Biotechnology (IBBA), Via Corti 12, 20133 Milan, Italy; sparvoli@ibba.cnr.it

**Keywords:** NMR, metabolomics, chemometrics, lactic acid bacteria, fermentation, legumes, common bean, *Phaseolus vulgaris*

## Abstract

In recent years, fermented foods have attracted increasing attention due to their important role in the human diet, since they supply beneficial health effects, providing important sources of nutrients. In this respect, a comprehensive characterization of the metabolite content in fermented foods is required to achieve a complete vision of physiological, microbiological, and functional traits. In the present preliminary study, the NMR-based metabolomic approach combined with chemometrics has been applied, for the first time, to investigate the metabolite content of *Phaseolus vulgaris* flour fermented by different lactic acid bacteria (LAB) and yeasts. A differentiation of microorganisms (LAB and yeasts), LAB metabolism (homo- and heterofermentative hexose fermentation), LAB genus (*Lactobacillus*, *Leuconostoc*, and *Pediococcus*), and novel genera (*Lacticaseibacillus*, *Lactiplantibacillus*, and *Lentilactobacillus*) was achieved. Moreover, our findings showed an increase of free amino acids and bioactive molecules, such as GABA, and a degradation of anti-nutritional compounds, such as raffinose and stachyose, confirming the beneficial effects of fermentation processes and the potential use of fermented flours in the production of healthy baking foods. Finally, among all microorganisms considered, the *Lactiplantibacillus plantarum* species was found to be the most effective in fermenting bean flour, as a larger amount of free amino acids were assessed in their analysis, denoting more intensive proteolytic activity.

## 1. Introduction

Innovations in the food industry are exploring improvement processes, such as fermentation, to develop novel functional foods from conventional or unconventional raw materials. Microbial fermentation is an ancient technology aimed to enhance shelf life, to provide new and peculiar flavors, and to improve the nutritional value of foods by transforming raw components into health-promoting compounds [1]. Yeasts and lactic acid bacteria (LAB) are widely used microorganisms in fermentation processes [2,3,4]. Carbohydrates are the substrate for yeasts and LAB to produce alcoholic [5] or lactic acid derivatives [3,6] as the end products of alcoholic or lactic fermentations, respectively. According to their metabolism, LAB can be distinguished into homo- or heterofermentative categories; homofermentation converts carbohydrates into lactate, while heterofermentation produces lactate, ethanol, acetate, and carbon dioxide in equimolar amounts [7]. In addition, early taxonomy based on morphology, carbon source as sugar, and growth temperature allow the classification of LAB into a few genera, including *Lactobacillus*, *Leuconostoc*, and *Pediococcus* [8,9]. Through the availability of DNA sequencing technologies, LAB classification has been extended, up to the recent reorganization of the *Lactobacillus* genus, into novel genera, among which *Lacticaseibacillus*, *Lactiplantibacillus*, and *Lentilactobacillus* [10] are included. Such classifications can be correlated to species-dependent profiles of fermentative metabolites, prone to be investigated with metabolomics [11,12]. 

Nuclear magnetic resonance (NMR) represents one of the most powerful and exhaustive approaches to monitor the metabolite content of complex matrices, providing both qualitative and quantitative evaluation of different classes of chemical compounds simultaneously. NMR data could be further analyzed by means of multivariate statistical analysis in order to detect possible sample differentiations/homologies according to specific metabolites, thus providing markers for different processes or treatments [13,14]. Some examples of NMR-based metabolomic studies of fermented foods and beverages are reported in the literature. Specifically, an NMR metabolomic approach was applied to monitor changes in metabolites in cheese ripening with added autochthonous LAB [15]; in milk fermentation for yogurt production [16]; in soymilk [17] and coconut milk [18] fermented with specific strains of bacteria; in fermented plant- and fruit-based juices to improve their functional properties [19], flavor [20], and shelf life [21]; and in fermented cereals to analyze the nutraceutical and antioxidant profile [22]. In the present work, NMR-based metabolomics, combined with multivariate statistical methods, was applied, for the first time, to analyze fermented common bean (*Phaseolus vulgaris*) flour. This flour is naturally enriched in proteins, dietary fibers, vitamins, minerals, and flavonoids but also characterized by the presence of some anti-nutritional factors (ANFs), such as toxic proteins like lectins that, if not properly heat inactivated, may cause adverse gastrointestinal effects, phytates that reduce mineral cation bioavailability, and raffinose family oligosaccharides (RFOs), represented by raffinose, stachyose, and verbascose, whose fermentation is the main cause of intestinal gases [23]. Due to the numerous nutraceutical benefits of this flour, we planned to exploit the advantages of fermentative processes, carried out by nine selected LAB and two yeasts, to reduce the ANF components like RFOs and to enrich the content of nutritional compounds. The fermented products were investigated by NMR and chemometrics, revealing a coherent strain differentiation based on metabolism- and genera-dependent fermentative metabolites. Moreover, the reduction in RFOs and an improvement in beneficial compounds, such as free amino acids (FAAs) and γ-aminobutyric acid (GABA), a bioactive compound known for its anti-hypertensive and antidepressant activities [24], were observed as the result of fermentation processes. 

## 2. Results and Discussion

### 2.1. Phaseolus vulgaris Flour Fermentation

The experimental design adopted in this work firstly involved a small-scale fermentation (ss) step using nine strains of LAB and two yeasts as starters (Table 1), accurately selected on the basis of their biochemical characteristics (exopolysaccharide synthesis, antimicrobial activity, carbon dioxide production, etc.), related to their class group. Non-inoculated bean flour was used as the control (CTRL). LAB fermentation resulted in a decrease of pH, due to the synthesis of organic acids. Yeast-fermented flour displayed a less acidic pH (Table 1), as yeasts mainly produce alcohols. 

Then, the small-scale protocol was scaled up to verify that productivity and quality were not affected by large-scale conditions, allowing for planning a potential use of this fermented flour for the preparation of baked products and providing useful information for applications in the food sector. In this respect, based on their biochemical characteristics and observed results in terms of RFO (raffinose and stachyose) reduction and GABA and FAA production, three LAB strains (*Lacticaseibacillus rhamnosus* LRH01, *Lentilactobacillus buchneri* LBC01, and *Leuconostoc lactis* LN01) and the two yeasts (*Kazachstania humilis* 2 and *Saccharomyces cerevisiae* 2B) were selected to perform large-scale fermentations (ls), up to a total of 2.5 kg of fermented product. After homogenizing the dough, a few grams of each sample were lyophilized to be analyzed.

### 2.2. NMR Spectra Analysis

The CTRL sample and the lyophilized products of fermentation were investigated by NMR, and the metabolite content (Table 2 and Figure S1) was determined on the basis of resonance assignments, by means of homo- and heteronuclear two-dimensional NMR spectra (TOCSY, HSQC, and HMBC) and with the aid of public databases (BMRB [25]).

NMR spectra analysis showed that, along with fermentative metabolites such as organic acids (lactate, acetate) and alcohols (ethanol, 2,3-butanediol), microbial fermentation of *Phaseolus vulgaris* flour produced some interesting bioactive compounds. As a matter of fact, the proteolytic activity of the selected microorganisms released FAAs, and among them, some essential amino acids, such as valine, leucine, and phenylalanine (whose presence represents an added value) [26], were detected in higher amount in fermented flours than in the CTRL sample. In addition, GABA content was found to be increased in fermented flours. These findings are supported by extensive literature reporting the potential of proteolytic activity and biosynthesis of health-beneficial compounds by microorganisms in fermentative processes [1,27,28,29,30]. 

### 2.3. Multivariate Statistical Analysis 

In order to evaluate the influence of the metabolites on sample separations, multivariate statistical analysis was applied to the complete ^1^H NMR spectra as well as to selected spectral regions, particularly the aliphatic and the aromatic ones. A total of 17 samples, which included the non-fermented bean flour control sample (CTRL), 12 LAB-fermented bean flour (hereafter LAB-fermented) samples, and four yeast-fermented bean flour (hereafter yeast-fermented) samples, in small and large scales (ss and ls), were considered.

**Figure 1:**
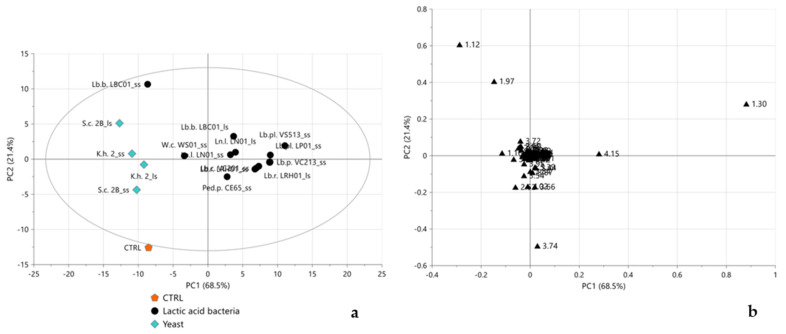
Score (**a**) and loading (**b**) plots of PCA performed considering all samples and the complete ^1^H NMR spectra. In (**a**), orange pentagons, black circles, and light blue diamonds represent CTRL, LAB-fermented, and yeast-fermented samples, respectively; in (**b**) black triangles represent the loadings of the variables of X matrix (the numbers refer to the initial ppm values of the buckets). 3PCs, R^2^X = 95.6%, Q^2^cum = 61.1%.

**Figure 2:**
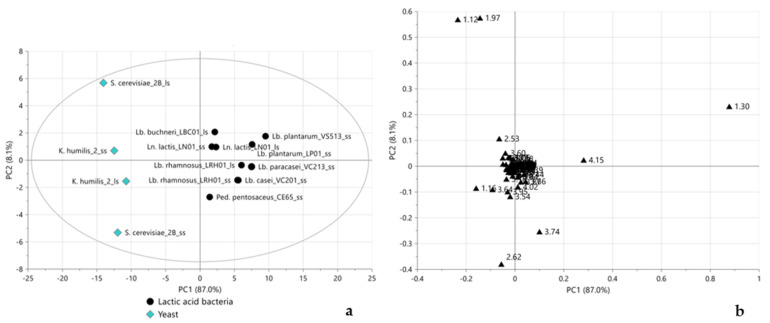
Score (**a**) and loading (**b**) plots of PCA performed, excluding CTRL, LBC01_ss, and WS01_ss samples and considering the complete ^1^H NMR spectra. In (**a**), black circles and light blue diamonds represent LAB-fermented and yeast-fermented samples, respectively; in (**b**) black triangles represent the loadings of the variables of X matrix (the numbers refer to the initial ppm values of the buckets). 2PCs, R^2^X = 95.1%, Q^2^cum = 70.7%.

The score plot of the PCA model performed on all samples, displayed in Figure 1a, showed LBC01_ss and WS01_ss well separated from all other LAB-fermented and yeast-fermented samples, clustering on the right and left sides of the score plot, respectively. This could be justified by a slightly lower amount of initial CFU/mL (<7.0 log), leading to an incomplete or slower fermentation process that determined a less acidic final pH than other LAB (Table 1). As expected, the CTRL sample, having experienced no fermentation process, was well separated from all the other samples, becoming as a moderate outlier. The corresponding loading plot (Figure 1b) indicated sucrose (bucket at 3.74 ppm) as the characteristic metabolite for the CTRL sample, while LAB-fermented flours, grouped on the right side of the score plot, were characterized by lactate (buckets at 1.30 and 4.15 ppm). LBC01_ss and WS01_ss samples were found to be characterized, as yeast-fermented samples, by acetate (bucket at 1.97 ppm) and 2,3-butanediol (bucket at 1.12 ppm). In order to better focus on the type of microorganism-, metabolism-, and genera-dependent fermentative metabolite differentiations, subsequent analyses were performed excluding the CTRL, LBC01_ss, and WS01_ss samples. The new PCA permitted observation of a much clearer separation between LAB-fermented and yeast-fermented flours along the first PC (Figure 2a). The corresponding loading plot (Figure 2b) highlighted lactate and sucrose and acetate, 2,3-butandiol, ethanol (buckets at 1.16 ppm and 3.64 ppm), citrate (bucket at 2.62 ppm), and acetoacetate (bucket at 2.53 ppm) as the characteristic metabolites for LAB-fermented and yeast-fermented samples, respectively. Citrate and 2,3-butanediol are two additional metabolites of fermentation, used in the food industry because of their flavoring activity when converted to diacetyl, an important preservative agent in foods due to its acidity [31,32,33].

Interestingly, samples in double scale (*Lb. rhamnosus*, *Ln. lactis*, *K. humilis*) seemed not to be affected by scaling up, except for *S. cerevisiae*, denoting, most likely, an anomalous fermentation process. Actually, prolonged fermentation (48 h) applied with *S. cerevisiae*, as a result of the high pH of the dough, could favor the development of alterative microorganisms naturally present in bean flour, affecting the stability of the dough and the reproducibility of the results [34].

**Figure 3:**
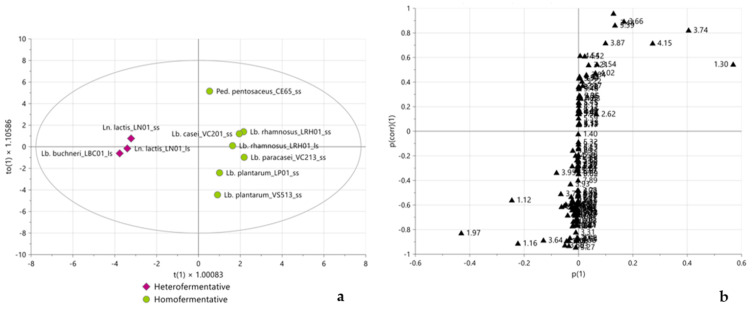
Score plot (**a**) and S-plot (**b**) of OPLS-DA performed according to LAB metabolism, considering the complete ^1^H NMR spectra. In (**a**), burgundy diamonds and green circles represent hetero- and homofermentative LAB-fermented samples, respectively; in (**b**) black triangles represent the weight of the variables of X matrix (the numbers refer to the initial ppm values of the buckets). One predictive and one orthogonal component, R^2^X = 79.7%, R^2^Y = 94.8%, Q^2^cum = 82.3%. Permutation tests are reported in Figure S2.

A supervised classification approach (OPLS-DA) was applied to LAB strains to maximize differentiations between homofermentative and heterofermentative glycolytic pathways. As depicted in the score plot in Figure 3a, the samples were clearly separated according to metabolism along the predictive component: homofermentative LAB clustered on the positive values of t1, while heterofermentative LAB clustered in the opposite direction. The corresponding S-plot, represented in Figure 3b, showed homofermentative LAB mainly characterized by lactate and sucrose, while heterofermentative LAB were mainly characterized by ethanol, acetate, and 2,3-butanediol.

**Figure 4:**
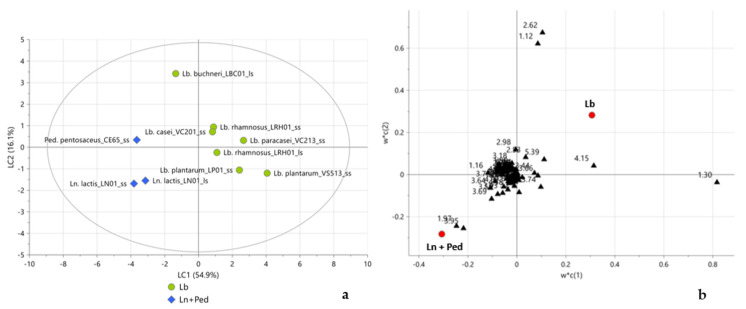
Score (**a**) and weight (**b**) plots of PLS-DA performed according to LAB genera in the early taxonomy, considering the complete ^1^H NMR spectra. In (**a**), green circles and blue diamonds represent the *Lactobacillus* genus and the *Leuconostoc* and *Pediococcus* genera, respectively; in (**b**) black triangles represent the weight of the variables of X matrix (the numbers refer to the initial ppm values of the buckets) while red circles represent the weight of the variables of Y matrix. 2LCs, R^2^X = 71%, R^2^Y = 93.5%, Q^2^cum = 63.3%. Permutation tests are reported in Figure S3.

Subsequently, PLS-DA was performed according to LAB genera as defined in the early taxonomy. The score plot (Figure 4a) showed a clear separation between the *Lactobacillus* genus and the *Leuconostoc* and *Pediococcus* genera along the first latent component. The corresponding weight plot (Figure 4b) showed lactate, citrate, and 2,3-butanediol as markers for the *Lactobacillus* genus and acetate and bucket at 3.95 ppm, not yet assigned, as characteristic metabolites for *Leuconostoc* and *Pediococcus* genera.

**Figure 5:**
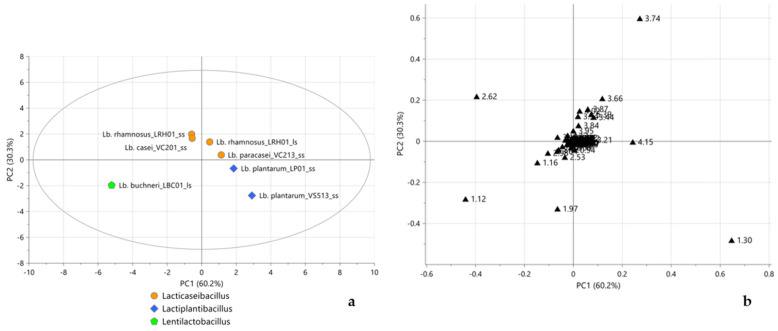
Score (**a**) and loading (**b**) plots of PCA performed on LAB-fermented flours considering only genera in the latest taxonomy and the complete ^1^H NMR spectra. In (**a**), orange circles, blue diamonds, and green pentagons represent *Lacticaseibacillus*, *Lactiplantibacillus*, and *Lentilactobacillus* samples, respectively; in (**b**) black triangles represent the loadings of the variables of X matrix (the numbers refer to the initial ppm values of the buckets). 3PCs, R^2^X = 97.6%, Q^2^cum = 74.7%.

Finally, in order to evaluate a possible sample differentiation according to the novel classes of genera, a PCA model was performed considering only flours fermented by the *Lactobacillus* genus bacteria. The score plot (Figure 5a) showed a clear separation between the three novel classes of genera described in the latest taxonomy 10. Actually, *Lacticaseibacillus* samples clustered in the upper part of the score plot, while *Lactiplantibacillus* and *Lentilactobacillus* grouped in the lower right and left sides of the score plot, respectively. As shown in the loading plot (Figure 5b), the compounds responsible for this separation were mainly fermentative metabolites, such as sucrose (buckets at 3.66, 3.74, and 5.39 ppm), lactate, ethanol, acetate, 2,3-butanediol, and citrate.

Particularly, as expected, as a consequence of heterofermentative metabolism, bean flour fermented by *Lentilactobacillus* bacteria resulted in being characterized by a higher content in ethanol, acetate, and 2,3-butanediol. A greater lactate production was instead observed for *Lactiplantibacillus (Lb. plantarum* strains), while citrate and sucrose characterized the other homofermentative *Lactobacillus* (*Lb. casei*, *Lb. paracasei*, and *Lb. rhamnosus*), highlighting that among the others, the *Lb. plantarum* species performed a more effective fermentative process. A plausible reason is that bean flour represented a suitable environment for those species rather than others. Further evidence for this assumption was provided by the multivariate statistical analysis performed on the aromatic and aliphatic regions of ^1^H NMR spectra, which also revealed other metabolites produced during fermentation, which were found to be characteristic of this genus. The PCA performed on the aliphatic (Figure S4) and aromatic (Figure S5) regions indicated *Lactiplantibacillus (Lb. plantarum* strains) characterized by both aliphatic FAAs, such as leucine (buckets at 0.94 and 1.62 ppm), valine (buckets at 0.98 and 1.03 ppm), methionine (bucket at 2.08 ppm), isoleucine (buckets at 0.90 and 1.01 ppm), and alanine (bucket at 1.47 ppm), and aromatic FAAs, such as phenylalanine (buckets at 7.21, 7.35 and 7.41 ppm), tyrosine (bucket at 7.15 ppm), and another aromatic compound, tyramine (buckets at 6.89 and 7.15 ppm). These findings demonstrated the most effective proteolytic activity in bean flour fermentation by these bacteria.

### 2.4. Essential Amino Acids, GABA, and RFO Analysis

Finally, the focus was placed on the improvements of fermented products in terms of beneficial compounds, such as the increase in FAA and GABA contents and the reduction in RFOs (raffinose and stachyose).

**Figure 6:**
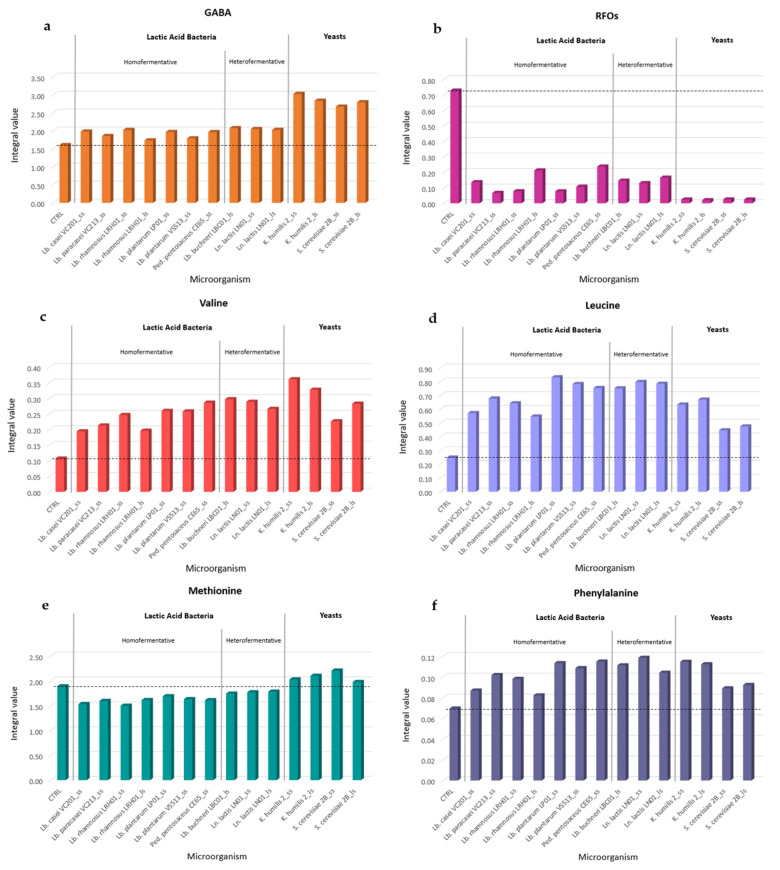
Quantification of the relative content (integral values normalized to the total spectral area) of metabolites of interest for nutritional purposes in small- (ss) and large-scale (ls) fermented bean flours (with the exclusion of LBC01_ss and WS01_ss samples) compared to control (CTRL). (**a**) GABA, (**b**) RFO, (**c**) Valine, (**d**) Leucine, (**e**) Methionine, and (**f**) Phenylalanine trends. The dotted line indicates the integral value, for each metabolite, in the CTRL sample.

For this purpose, the evolution of GABA, RFOs, and some essential amino acids, such as valine, leucine, methionine, and phenylalanine, was investigated (Figure 6a–f). The selection of the above-mentioned essential amino acids was driven by the possibility of integrating the corresponding isolated signals in the NMR spectra. In addition, the evolution of fermentative metabolites, such as lactate, ethanol, acetate, 2,3-butanediol, and sucrose, was also analyzed and reported in Figure S6a–e.

As showed in Figure 6a, GABA content increased in all fermented flours compared with CTRL, achieving a higher relative content when fermentation was carried out by yeasts. RFO degradation occurred strongly in all fermented products, being almost complete when fermentation was carried out by yeasts (Figure 6b). The content of valine (Figure 6c), leucine (Figure 6d), and phenylalanine (Figure 6f) showed an increment in all fermented flours compared with the CTRL sample. Particularly, valine content was higher in yeast- and heterofermentative LAB-fermented flours; leucine content was higher in LAB- and *K. humilis* yeast-fermented samples and reached the lowest amount in *S. cerevisiae* yeast-fermented samples; phenylalanine showed an almost similar increment in all samples (Figure 6f). Interestingly, methionine content (Figure 6e) was higher than CTRL, albeit slightly, only in yeast-fermented flour samples; in LAB-fermented flours, its content decreased, achieving the lowest values in homofermentative LAB samples. A similar trend of these metabolites was reported in a previous study, in which nineteen traditional Italian legumes, including eight different bean flours belonging to *Phaseolus vulgaris*, were fermented by *Lactobacillus plantarum* C48 and *Lactobacillus brevis* AM7 and subsequently analyzed by various approaches [35]. The advantage of using the NMR spectroscopy, as proposed in the present study, is the possibility to obtain, within a single experiment, all the metabolite information simultaneously, otherwise achievable by performing several analyses. Moreover, this high-throughput method was suitable for screening different microorganisms (LAB and yeasts) and comparing them on the basis of the metabolites produced by fermentation. Among the LAB, the *Lb. plantarum* species, along with *Lb. paracasei*, was found to be one of the most effective in decreasing RFO content during fermentation (Figure 6b). In agreement, the literature reports that the species *Lb. plantarum* was found to be effective in reducing RFOs when applied to ferment whole red haricot [36] and chickpea flours [37]. An increment in essential amino acids in fermented bean flours (*Phaseolus vulgaris*) was also observed when fermented by other types of microorganisms, such as *Pleurotus ostreatus* [38] and *Aspergillus oryzae* [39] fungi. These findings suggest that fermentation, carried out by the considered microorganisms on bean flour, results in a final product with improved nutritional properties. As a matter of fact, the reduction of some anti-nutritional factors such as RFO content and the production of bioactive compounds such as GABA and some essential amino acids, in different ratios according to the microorganism considered, were observed.

Figure S6a–e shows the trend of metabolites mainly involved in fermentation processes. It is noteworthy that each class of microorganisms displayed a peculiar composition in the production of fermentative metabolites, strictly related to their classification, with a clear differentiation between LAB and yeasts and between homo- and heterofermentative metabolism of LAB. As expected, sucrose was found to be highly consumed by yeasts, producing higher ethanol content. Moreover, yeasts showed the highest production of 2,3-butanediol along with *L. buchneri*_LBC01_ls. As expected, the other microorganisms did not produce significant amounts of 2,3-butanediol, as it is known that the biosynthesis of 2,3-butanediol by bacteria occurs through mixed acid fermentation [7]. Sucrose, lactate, and ethanol trends in LAB-fermented samples perfectly reflected the different metabolism carried out by homo- and heterofermentative bacteria. Finally, the acetate content was higher in all fermented flours than in CTRL, achieving the highest amount in samples fermented with heterofermentative LAB and yeasts.

## 3. Materials and Methods

### 3.1. Microbial Strains

*Lacticaseibacillus casei* VC201, *Kazachstania humilis* 2, and *Saccharomyces cerevisiae* 2B used as fermentation starters belong to the CNR ISPA collection. *Lactiplantibacillus plantarum* ITEM 17219 (VS513), *Lacticaseibacillus paracasei* ITEM 17217 (VC213), and *Pediococcus pentosaceus* ITEM 18337 (CE65) were previously isolated from CNR ISPA and deposited in the Agro-Food Microbial Culture Collection of the Institute of Sciences of Food Production, CNR, Bari, Italy. *Weissella confuse* WS01, *Lentilactobacillus buchneri* LBC01, *Lacticaseibacillus rhamnosus* LRH01, *Lactiplantibacillus plantarum* LP01, and *Leuconostoc lactis* LN01 were provided by Sacco srl (Cadorago, Como, Italy). Each LAB strain was routinely propagated at 30 °C in de Man—Rogosa—Sharpe (MRS) broth (Biolife Italiana, Milan, Italy) for 18 h. Yeast strains were propagated at room temperature in Yeast Malt (YM) Broth (Biolife Italiana) for 24 h.

### 3.2. Fermentation Protocols

Bean (*Phaseolus vulgaris*) flour (Taylor’s horticultural variety kindly provided by Bernardi Corrado) fermentation was performed in small scale (100 g), with all described strains singly added. Among the microbial strains adopted in the small-scaled fermentation process, LBC01, LN01, and LRH01 bacteria and the two yeasts were selected, on the basis of NMR data analysis, to perform the scale-up of the fermentation process (2500 g) because of their good results in terms of GABA and FAA production and RFO reduction, as well as for their biochemical properties, such as heterofermentative metabolism, which represents an advantage in baked products due to CO_2_ release.

#### 3.2.1. Small-Scale Fermentation Process

For each strain, 1 mL of cultivated bacteria was centrifuged at 10,000× *g* for 10 min (Centrifuge 5425 Eppendorf AG, Hamburg, Germany), and the pellet was washed once with quarter-strength Ringer’s solution and centrifuged (Sorvall superspeed centrifuge RC2-B, Ivan Sorvall Inc., Norwalk, CT, USA). After discharging the supernatant, the pellet was resuspended in 40 mL of sterile tap water. For cell counting before fermentation, 1 mL of inoculated tap water was serially diluted in quarter-strength Ringer’s solution (Scharlau Microbiology, Barcelona, Spain) and plated on MRS agar for LAB or Chloramphenicol Glucose Agar (CGA) (Scharlau Microbiology) for yeasts. LAB plates were incubated at 30 °C for 48 h under anaerobic conditions (AnaerocultA, Merck, Darmstad, Germany), while yeast plates were incubated at 25 °C for five days in aerobic conditions. Dough was prepared by mixing 40 g of bean flour and 60 mL of sterilized tap water, previously inoculated at ca. 7.0 log CFU/mL. After 48 h of incubation at 30 °C, each species increased by 2 log cycles, determining a species-dependent pH variation compared to the control pH value of 6.50. The pH value was determined using a pH meter (HI 122, HANNA Instruments, Ronchi di Villafranca Padovana, Italy). For microbial enumeration after fermentation, 5 g of the fermented product were homogenized in 45 mL of a 2% (*w*/*v*) sterile Buffered Peptone Water (Oxoid LTD., Basingstoke, Hampshire, UK) for 2 min in a Stomacher BagMixer (Interscience, St. Nom, France). The sample was serially diluted in quarter-strength Ringer’s solution and plated on MRS agar supplemented with 0.1 g/L cycloheximide (Sigma-Aldrich, St. Louis, MO, USA) for LAB or YGC for yeasts. LAB plates were incubated at 30 °C for 48 h under anaerobic conditions, while yeast plates were incubated at 25 °C for five days in aerobic conditions. After homogenizing the dough, 2–3 g were sampled to be lyophilized.

#### 3.2.2. Large-Scale Fermentation Process

Twenty mL of the selected cultivated microorganisms were centrifuged at 6000× *g* for 10 min. After washing the pellet with quarter-strength Ringer’s solution and centrifuging, the pellet was resuspended in 1.5 L of sterile tap water. Starting cells were counted as described before. A lab-scale kneading device (Artisan 5KSM150PS KitchenAid, St. Joseph, MI, USA) equipped with a hook was used to knead 1 kg of bean flour and 2.5 L of inoculated tap water. Dough was incubated at 30 °C for 48 h for fermentation. pH and final cell counting in the fresh fermented product were determined as described above. After homogenizing the dough, 2–3 g were sampled to be lyophilized.

Table 1 reports all the microbial starters employed, with details concerning the LAB classification and the fermentation conditions.

### 3.3. NMR Analysis

#### 3.3.1. Sample Preparation

Fifty mg of homogenized bean flour (control, CTRL) or lyophilized fermented flours were mixed vigorously with 750 µL of phosphate buffer (1 M, pH 4.16 ± 0.09) in D_2_O (Sigma-Aldrich, St. Louis, MO, USA) and incubated at room temperature for 30 min. After centrifugation at 12,100× *g* for 7 min (Centrifuge MiniSpin^®^ Eppendorf, Hamburg, Germany), 600 µL of the aqueous supernatant were transferred in 5 mm NMR tubes. The pH values were checked for all samples, resulting in good agreement. The CTRL sample was checked for changes in metabolite composition over time by ^1^H NMR, showing a robust stability.

#### 3.3.2. NMR Data Acquisition and Processing

All NMR spectra were acquired at 300 K on a Bruker Advance Neo 600 spectrometer (Bruker Biospin, GmbH Rheinstetten, Karlsruhe, Germany) operating at 14.07 T, equipped with a 5 mm reverse Z gradient cryoprobe PRODIGY and a thermostatted autosampler.

^1^H NMR spectra were acquired with a monodimensional NOESY sequence with a solvent presaturation scheme with 256 scans over 64 K points of data and a spectral width of 7800 Hz. A total relaxation time of 19.2 s was used to allow a complete relaxation of all nuclei.

A resolution enhancement function (LB = 0.3 Hz) was applied before Fourier transformation. All spectra were phased, baseline corrected, and aligned with respect to a uracil signal at 5.81 ppm (TOPSPIN software version 4.1.2, Bruker Biospin, GmbH Rheinstetten, Karlsruhe, Germany). After the exclusion of residual solvent regions between 4.71–4.97 ppm, the spectra were subjected to intelligent bucketing in the range of 0.70–10.00 ppm according to the resonance assignment, and integrals were normalized to the total spectral area using the ACD/NMR software (ACD Labs, version 11, Toronto, ON, Canada).

In addition, the aromatic (5.76–10.00 ppm) and aliphatic regions (0.70–3.44 ppm), after the exclusion of 2,3-butanediol and ethanol (1.11–1.20 ppm), acetate (1.97–2.08 ppm), and lactate (1.30–1.40 ppm) signals, were considered independently to evaluate the contributions of specific aliphatic and aromatic signals to class separation. For each new dataset, integrals were normalized to the total spectral area considered.

Bidimensional TOCSY, HSQC, and HMBC spectra were recorded with the same spectral parameters of the monodimensional spectra, with 256 scans, by using the non-uniform sampling acquisition method.

### 3.4. Statistical Methods

NMR data were imported into SIMCA-P 17.0.2 (Sartorius Data Analytics, Umeå, Sweden) for multivariate statistical analysis. Principal component analysis (PCA), projection to latent structures discriminant analysis (PLS-DA), and orthogonal projection to latent structures discriminant analysis (OPLS-DA) were performed by using “mean centering” as data pretreatment. To overcome randomness of PLS-DA and OPLS-DA models, the permutation test was checked for each model.

## 4. Conclusions

Fermentations with microorganisms are processes widely applied in the food industry to improve the nutritional properties, flavor, and stability of foods. During this process, food components are consumed and converted into new metabolites, favoring the reduction of some anti-nutritional factors and/or the production of new bioactive compounds. In this work, a screening of LAB and yeasts in the fermentation of common bean (*Phaseolus vulgaris*) flour was carried out with both small- and large-scale protocols, and, for the first time, was investigated by NMR-based metabolomics and multivariate statistical methods. The results showed clear differentiations, mainly driven by fermentative metabolites (lactate, ethanol, 2,3-butanediol, acetate, citrate), according to fermentative microorganisms (LAB and yeasts), LAB metabolism (homo- and heterofermentation), LAB genera (*Lactobacillus* and *Leuconostoc*/*Pediococcus*), and novel *Lactobacillus* genera (*Lacticaseibacillus*, *Lactiplantibacillus*, and *Lentilactobacillus*). In addition, these studies revealed that the *Lb. plantarum* species was the most effective in fermenting bean flour, as larger amounts of FAAs were detected in their analysis, denoting more intensive proteolytic activity. Regarding the reduction of anti-nutritional components, the results showed extensive or even complete degradation of RFO content after the fermentation process; conversely, GABA content increased in all fermented flours. In conclusion, notwithstanding the preliminarily nature of this study, it provided useful and valid information in screening LAB and yeasts in common bean (*Phaseolus vulgaris*) flour fermentation. The metabolite profiling, performed by ^1^H NMR, combined with multivariate statistical analysis suggested that this treatment resulted in an excellent new ingredient to produce more nutrient-composite flours. Interestingly, the scale-up of the fermentation process did not affect the production of metabolites for *Lb. rhamnosus*, *Ln. lactis*, and *K. humilis* strains, thus suggesting the feasibility of large-scale fermentations that can be adapted for the food industry. In fact, fermented bean flour could be usefully employed to replace an extensive portion of wheat flour in the production of baked goods, becoming a vehicle for nutrient intake. In this regard, very recently, successful results were achieved in the production of cookies [40] and bread [41], fermenting oat and bean flours by fungi.

## Figures and Tables

**Table 1:** Microbial starters selected for *Phaseolus vulgaris* flour fermentation and their discussed classifications. Scale, initial and final cell counting, and pH values after incubation of fermented flours are reported.

Sample	Microorganism	Hexose Fermentation	Early Taxonomy Genera	Latest Taxonomy Genera	Scale Small (ss)/Large (ls)	Initial Cell Counting (10^7^ CFU/mL)	Final Cell Counting (10^9^ CFU/g)	pH
*Lacticaseibacillus casei* VC201	LAB	Homofermentative	*Lactobacillus*	*Lacticaseibacillus*	ss	5.1	5.8	4.20
*Lacticaseibacillus paracasei* VC213	LAB	Homofermentative	*Lactobacillus*	*Lacticaseibacillus*	ss	12	15.5	4.17
*Lactiplantibacillus plantarum* LP01	LAB	Homofermentative	*Lactobacillus*	*Lactiplantibacillus*	ss	7.8	2.8	4.18
*Lactiplantibacillus plantarum* VS513	LAB	Homofermentative	*Lactobacillus*	*Lactiplantibacillus*	ss	14.5	10.4	4.21
*Lacticaseibacillus rhamnosus* LRH01	LAB	Homofermentative	*Lactobacillus*	*Lacticaseibacillus*	ss/ls	6/7.8	7.4/2.6	4.29/4.41
*Lentilactobacillus buchneri* LBC01	LAB	Heterofermentative	*Lactobacillus*	*Lentilactobacillus*	ss/ls	0.1/1.2	0.12/2.7	4.83/4.23
*Leuconostoc lactis* LN01	LAB	Heterofermentative	*Leuconostoc*	*Leuconostoc*	ss/ls	2.1/1.3	4.1/7.6	4.22/4.48
*Pediococcus pentosaceus* CE65	LAB	Homofermentative	*Pediococcus*	*Pediococcus*	ss	7.2	4.6	4.55
*Weissella confusa* WS01	LAB	Heterofermentative	*Weissella*	*Weissella*	ss	1	3.3	5.14
*Kazachstania humilis 2*	Yeast	-	-	-	ss/ls	8.6/3.8	0.40/0.25	6.34/6.04
*Saccharomyces cerevisiae 2B*	Yeast	-	-	-	ss/ls	1.7/2.6	0.11/0.18	6.35/6.18

**Table 2:** ^1^H-NMR metabolite assignments of aqueous extract of CTRL and fermented bean flours.

Peak	Compound	Assignment	ppm
1	2,3-Butanediol	CH_3_	1.14
2	Acetate	CH_3_	2.01
3	Acetoacetate	CH_3_	2.54
4	Acetoin	CH_3_	2.23, 1.38
5	Adenine	aromatics	8.24, 8.21
6	Alanine	βCH_3_	1.48
7	Asparagine	β/β′CH_2_	2.96, 2.89
8	Citrate	CH_2_	2.81, 2.69
9	Ethanol	CH_2_	3.65
		CH_3_	1.18
10	Formate	HCOO	8.43
11	GABA	all CH_2_	3.00, 2.38, 1.94
12	Galactose	αH1	5.27
		βH1	4.60
13	Glucose	αH1	5.25
		βH1	4.66
14	Isoleucine	γCH_3_	1.01
15	Lactate	CH	4.20
		CH_3_	1.35
16	Leucine	βCH_2_	1.70
		δ/δ′CH_3_	0.96
17	Methionine	S-CH_3_	2.13
18	Phenylalanine	aromatics	7.32–7.44
		β/β′CH_2_	3.22, 3.25
19	Pipecolic acid	all CH_2_	3.57, 3.41, 3.00, 2.21, 1.87, 1.63
20	Raffinose	H1α Glu	5.44
21	Stachyose	H1α Glu	5.43
22	Sucrose	H1α Glu	5.41
23	Trigonelline	aromatics	9.12, 8.84, 8.09
		N-CH_3_	4.44
24	Tyramine	aromatics	7.20, 6.90
25	Tyrosine	aromatics	7.18, 6.87
26	Uracil	aromatics	7.55, 5.81
27	Uridine	aromatics	7.86, 5.92
28	Valine	αCH	3.61
		γ, γ′CH_3_	0.99, 1.04

## Data Availability

Some data is contained within the article or Supplementary Material.

## Supplementary Materials

The following supporting information can be downloaded at https://www.mdpi.com/article/10.3390/molecules28124864/s1. Figure S1. ^1^H NMR spectra of aqueous extracts of all small-scale fermented bean flour samples compared to control (CTRL). The numbers refer to metabolite peak assignments as reported in Table 2. Figure S2. Permutation tests for OPLS-DA performed, according to LAB metabolism, considering the complete ^1^H NMR spectra, and 200 rounds of random permutations for each class: (a) hetero- and (b) homofermentative LAB-fermented samples. Figure S3. Permutation tests relative to PLS-DA performed according to LAB genera in the early taxonomy, considering the complete ^1^H NMR spectra, and 200 rounds of random permutations for each class: (a) *Leuconostoc* and *Pediococcus* genera and (b) *Lactobacillus* genus. Figure S4. Score (a) and loading (b) plots of PCA performed on LAB-fermented flours considering only genera in the latest taxonomy and the aliphatic region of ^1^H NMR spectra. In (a), orange circles, blue diamonds, and green pentagons represent *Lacticaseibacillus*, *Lactiplantibacillus*, and *Lentilactobacillus* samples, respectively; 2PCs, R^2^X = 96.1%, Q^2^cum = 71.2%. Figure S5. Score (a) and loading (b) plots of PCA performed on LAB-fermented flours considering only genera in the latest taxonomy and the aromatic region of ^1^H NMR spectra. In (a), orange circles, blue diamonds, and green pentagons represent *Lacticaseibacillus, Lactiplantibacillus*, and *Lentilactobacillus* samples, respectively; 3PCs, R^2^X = 89.9%, Q^2^cum = 30.8%. Figure S6. Quantification of the relative content (integral values normalized to the total spectral area) of fermentative metabolites: (a) lactate, (b) ethanol, (c) acetate, (d) 2,3-butanediol, and € sucrose in small- (ss) and large-scale (ls) fermented bean flour (with the exclusion of LBC01_ss, and WS01_ss samples) compared to control (CTRL). The dotted line indicates the initial content of each metabolite in the CTRL sample.

## References

[1] Şanlier N., Gökcen B.B., Sezgin A.C. (2019). Health benefits of fermented foods. Crit. Rev. Food Sci. Nutr..

[2] Rai A.K., Jeyaram K., Satyanarayana T., Kunze G. (2017). Role of Yeasts in Food Fermentation. Yeast Diversity in Human Welfare.

[3] Bintsis T. (2018). Lactic acid bacteria as starter cultures: An update in their metabolism and genetics. AIMS Microbiol..

[4] Castellone V., Bancalari E., Rubert J., Gatti M., Neviani E., Bottari B. (2021). Eating fermented: Health benefits of LAB-fermented foods. Foods.

[5] Maicas S. (2020). The Role of Yeasts in Fermentation Processes. Microorganisms.

[6] Wang Y., Wu J., Lv M., Shao Z., Hungwe M., Wang J., Bai X., Xie J., Wang Y., Geng W. (2021). Metabolism characteristics of lactic acid bacteria and the expanding applications in food industry. Front. Bioeng. Biotechnol..

[7] Kandler O. (1983). Carbohydrate metabolism in lactic acid bacteria. Antonie Van Leeuwenhoek.

[8] Orla-Jensen S. (1919). The Lactic Acid Bacteria.

[9] Axelsson L., Salminen S., von Wright A. (1998). Lactic acid bacteria: Classification and physiology. Lactic Acid Bacteria: Microbiology and Functional Aspects.

[10] Zheng J., Wittouck S., Salvetti E., Franz C.M.A.P., Harris H.M.B., Mattarelli P., O’Toole P.W., Pot B., Vandamme P., Walter J. (2020). A taxonomic note on the genus *Lactobacillus*: Description of 23 novel genera, emended description of the genus *Lactobacillus* Beijerinck 1901, and union of *Lactobacillaceae* and *Leuconostocaceae*. Int. J. Syst. Evol. Microbiol..

[11] Mozzi F., Ortiz M.E., Bleckwedel J., De Vuyst L., Pescuma M. (2013). Metabolomics as a tool for the comprehensive understanding of fermented and functional foods with lactic acid bacteria. Food Res. Int..

[12] Gao Y., Hou L., Gao J., Li D., Tian Z., Fan B., Wang F., Li S. (2021). Metabolomics approaches for the comprehensive evaluation of fermented foods: A review. Foods.

[13] Qu Q., Jin L. (2022). Application of nuclear magnetic resonance in food analysis. Food Sci. Tech. Brazil.

[14] Sundekilde U.K., Eggers N., Bertram H.C., Gowda G., Raftery D. (2019). NMR-Based Metabolomics of Food. NMR-Based Metabolomics.

[15] Piras C., Marincola F.C., Savorani F., Engelsen S.B., Cosentino S., Viale S., Pisano M.B. (2013). A NMR metabolomics study of the ripening process of the Fiore Sardo cheese produced with autochthonous adjunct cultures. Food Chem..

[16] Trimigno A., Bøge Lyndgaard C., Atladóttir G.A., Aru V., Balling Engelsen S., Harder Clemmensen L.K. (2020). An NMR metabolomics approach to investigate factors affecting the yoghurt fermentation process and quality. Metabolites.

[17] Yang S.-O., Kim S.-H., Cho S., Lee J.H., Kim Y.-S., Yun S.-S., Choi H.-K. (2009). Classification of fermented soymilk during fermentation by ^1^H NMR coupled with principal component analysis and elucidation of free-radical scavenging activities. Biosci. Biotechnol. Biochem..

[18] Qadi W.S.M., Mediani A., Benchoula K., Wong E.H., Misnan N.M., Sani N.A. (2023). Characterization of physicochemical, biological, and chemical changes associated with coconut milk fermentation and correlation revealed by ^1^H NMR-based metabolomics. Foods.

[19] Tomita S., Saito K., Nakamura T., Sekiyama Y., Kikuchi J. (2017). Rapid discrimination of strain-dependent fermentation characteristics among *Lactobacillus* strains by NMR-based metabolomics of fermented vegetable juice. PLoS ONE.

[20] Markkinen N., Pariyani R., Jokioja J., Kortesniemi M., Laaksonen O., Yang B. (2022). NMR-based metabolomics approach on optimization of malolactic fermentation of sea buckthorn juice with *Lactiplantibacillus plantarum*. Food Chem..

[21] Muhialdin B.J., Kadum H., Hussin A.S.M. (2021). Metabolomics profiling of fermented cantaloupe juice and the potential application to extend the shelf life of fresh cantaloupe juice for six months at 8 °C. Food Control.

[22] Colosimo R., Gabriele M., Cifelli M., Longo V., Domenici V., Pucci L. (2020). The effect of sourdough fermentation on *Triticum dicoccum* from Garfagnana: ^1^H NMR characterization and analysis of the antioxidant activity. Food Chem..

[23] Sparvoli F., Bollini R., Cominelli E., De Ron A. (2015). Nutritional value. Grain Legumes.

[24] Cui Y., Miao K., Niyaphorn S., Qu X. (2020). Production of gamma-aminobutyric acid from lactic acid bacteria: A systematic review. Int. J. Mol. Sci..

[25] BMRB—Biological Magnetic Resonance Data Bank. https://bmrb.io.

[26] Hou Y., Wu G. (2018). Nutritionally essential amino acids. Adv. Nutr..

[27] Corsetti A., Settanni L. (2007). Lactobacilli in sourdough fermentation. Food Res. Int..

[28] Melini F., Melini V., Luziatelli F., Ficca A.G., Ruzzi M. (2019). Health-promoting components in fermented foods: An up-to-date systematic review. Nutrients.

[29] Garrido-Galand S., Asensio-Grau A., Calvo-Lerma J., Heredia A., Andrés A. (2021). The potential of fermentation on nutritional and technological improvement of cereal and legume flours: A review. Food Res. Int..

[30] Emkani M., Oliete B., Saurel R. (2022). Effect of lactic acid fermentation on legume protein properties, a review. Fermentation.

[31] Ji X.-J., Huang H., Ouyang P.-K. (2011). Microbial 2,3-butanediol production: A state-of-the-art review. Biotechnol. Adv..

[32] Song C.W., Park J.M., Chung S.C., Lee S.Y., Song H. (2019). Microbial production of 2,3-butanediol for industrial applications. J. Ind. Microbiol. Biotechnol..

[33] Behera B.C., Mishra R., Mohapatra S. (2021). Microbial citric acid: Production, properties, application, and future perspectives. Food Front..

[34] Minervini F., De Angelis M., Di Cagno R., Gobbetti M. (2014). Ecological parameters influencing microbial diversity and stability of traditional sourdough. Int. J. Food Microbiol..

[35] Curiel J.A., Coda R., Centomani I., Summo C., Gobbetti M., Rizzello C.G. (2015). Exploitation of the nutritional and functional characteristics of traditional Italian legumes: The potential of sourdough fermentation. Int. J. Food Microbiol..

[36] Kitum V.C., Kinyanjui P.K., Mathara J.M., Sila D.N. (2020). Effect of *Lb. plantarum* BFE 5092 fermentation on antinutrient and oligosaccharide composition of whole red haricot bean (*Phaseolus vulgaris* L.). Int. J. Food Sci..

[37] Galli V., Venturi M., Pini N., Guerrini S., Granchi L. (2019). Exploitation of sourdough lactic acid bacteria to reduce raffinose family oligosaccharides (RFOs) content in breads enriched with chickpea flour. Eur. Food Res. Technol..

[38] Espinosa-Páez E., Alanis-Guzmán M.G., Hernández-Luna C.E., Báez-González J.G., Amaya-Guerra C.A., Andrés-Grau A.M. (2017). Increasing antioxidant activity and protein digestibility in *Phaseolus vulgaris* and *Avena sativa* by fermentation with the *Pleurotus ostreatus* fungus. Molecules.

[39] Lee Y.H., Lee N.-R., Lee C.H. (2022). Comprehensive metabolite profiling of four different beans fermented by *Aspergillus oryzae*. Molecules.

[40] Espinosa-Páez E., Hernández-Luna C.E., Longoria-García S., Martínez-Silva P.A., Ortiz-Rodríguez I., Villarreal-Vera M.T., Cantú-Saldaña C.M. (2021). *Pleurotus ostreatus*: A potential concurrent biotransformation agent/ingredient on development of functional foods (cookies). LWT.

[41] Espinosa-Páez E., Hernández-Luna C.E., Longoria-García S., Torres-Alvarez C., Velez-Argumedo C., González-Martínez B.E. (2023). Improving nutritional and functional quality charateristics in bread by using flours obtained from fermentation of kidney beans and oats with *Pleurotus ostreatus*. CYTA J. Food.

