# Supplementary material for: NMR-Based Metabolomic Study on Phaseolus vulgaris Flour Fermented by Lactic Acid Bacteria and Yeasts
Source: Molecules. 2023 Jun 20;28(12):4864. doi: 10.3390/molecules28124864 (PMC10302956; doi:10.3390/molecules28124864)
Supplement: Supplementary file 1 [file molecules-28-04864-s001.zip › molecules-2410035-supplementary_PROOF.pdf]

Supplementary materials

NMR-based metabolomic study on *Phaseolus vulgaris* flour fermented by lactic acid bacteria and yeasts

Giuseppina Tatulli [1], Laura Ruth Cagliani [2,*], Francesca Sparvoli [3], Milena Brasca [1], and Roberto Consonni [2]

[1] National Research Council, Institute of Sciences of Food Production (ISPA), via Celoria 2, 20133, Milan, Italy
[2] National Research Council, Institute of Chemical Sciences and Technologies "G. Natta" (SCITEC), via Corti 12, 20133, Milan, Italy
[3] National Research Council, Institute of Agricultural Biology and Biotechnology (IBBA), via Corti 12, 20133, Milan, Italy
* Correspondence: lauraruth.cagliani@scitec.cnr.it; Tel. +390223699722

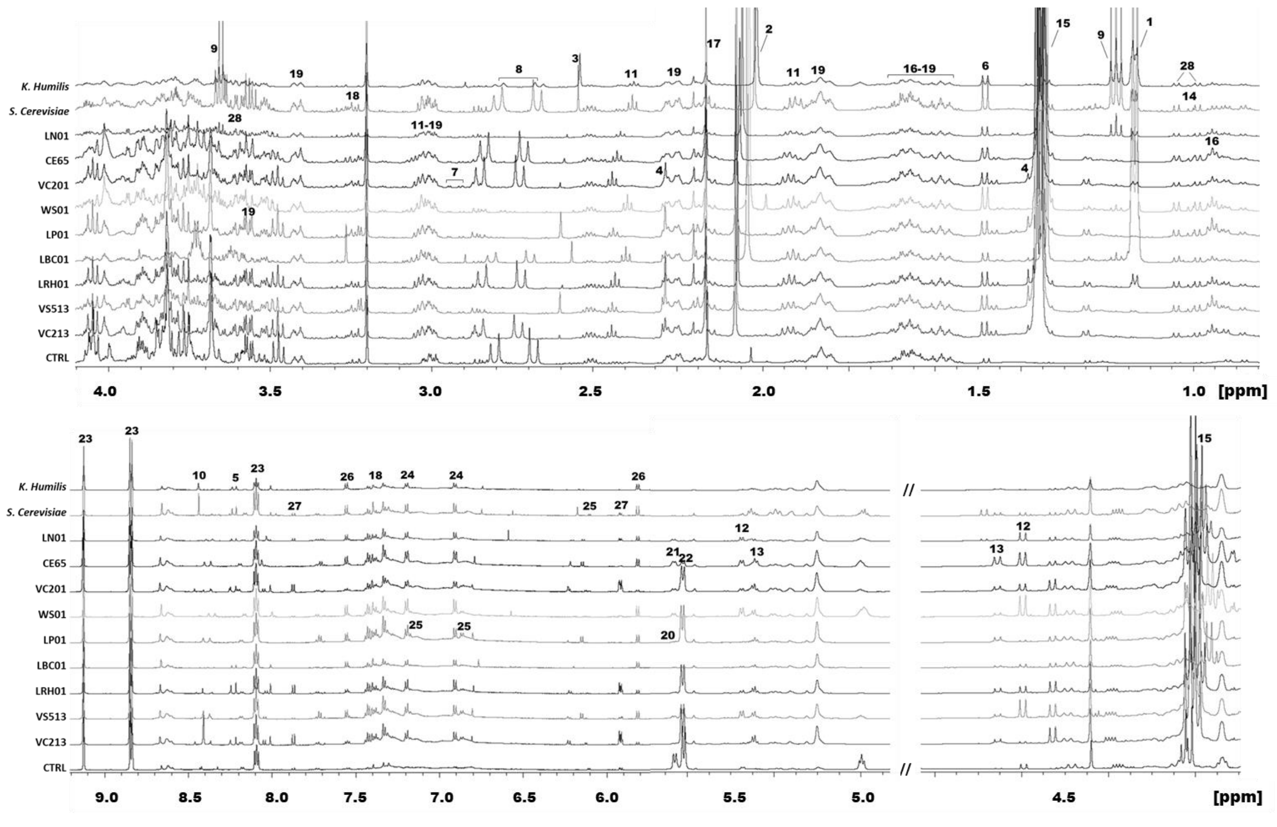

Figure S1. ^{1}H NMR spectra of aqueous extracts of all small-scale fermented bean flour samples, compared to control (CTRL). The numbers refer to metabolite peak assignments, as reported in Table 2.

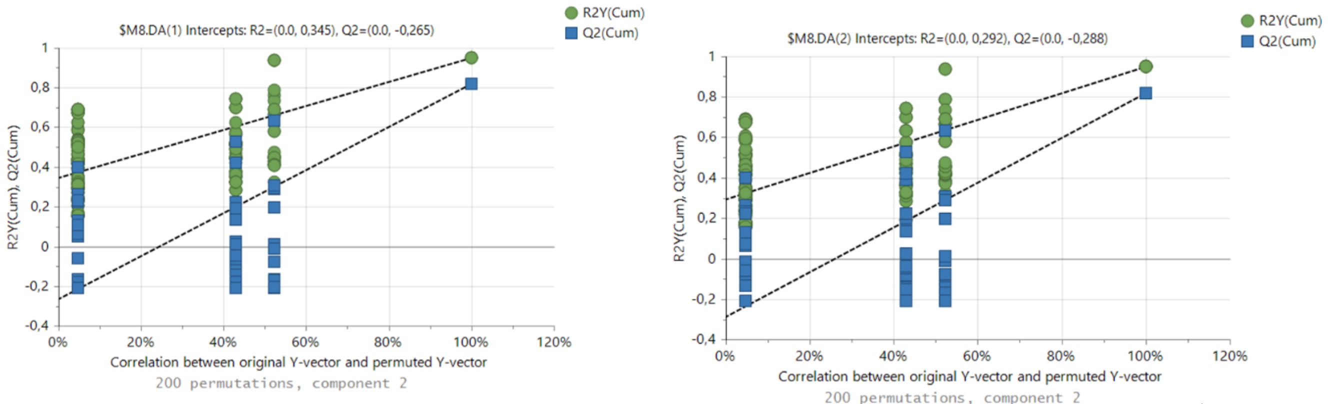

a **b**

Figure S2. Permutation tests for OPLS-DA performed according to LAB metabolism, considering the complete ^{1}H NMR spectra, and 200 rounds of random permutations for each class: **a)** hetero- and **b)** homofermentative LAB-fermented samples.

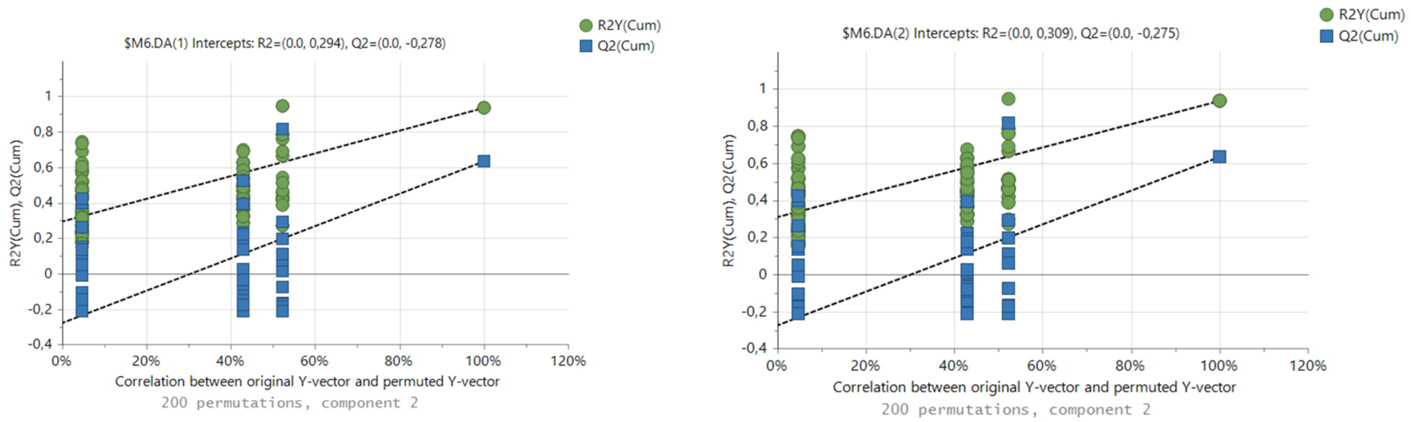

a **b**

Figure S3. Permutation tests relative to PLS-DA performed according to LAB genera in the early taxonomy, considering the complete ^{1}H NMR spectra, and 200 rounds of random permutations for each class: **a)** *Leuconostoc* and *Pediococcus* genera, and **b)** *Lactobacillus* genus.

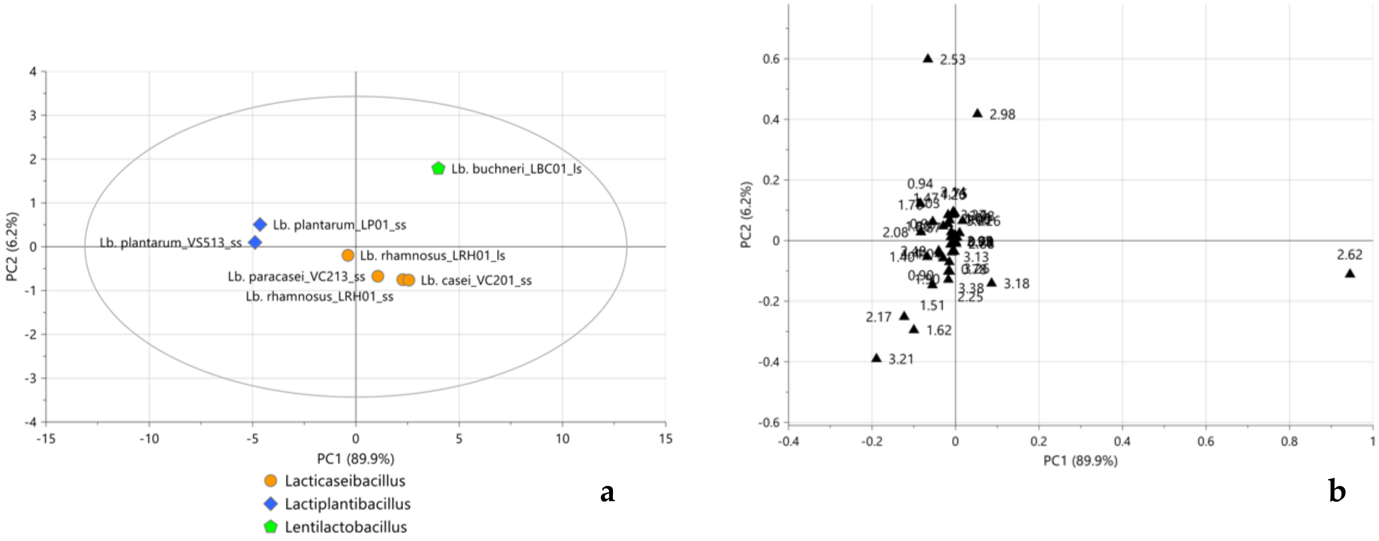

Figure S4. Score **(a)** and loading **(b)** plots of PCA performed on LAB-fermented flours, considering only genera in the latest taxonomy and the aliphatic region of ^{1}H NMR spectra. In **(a)**, orange circles, blue diamonds, and green pentagons represent *Lacticaseibacillus*, *Lactiplantibacillus,* and *Lentilactobacillus* samples, respectively; in **(b)** black triangles represent the loadings of the variables of X matrix (the numbers refer to the initial ppm values of the buckets). 2PCs, R^2X=96.1%, Q^2cum= 71.2%.

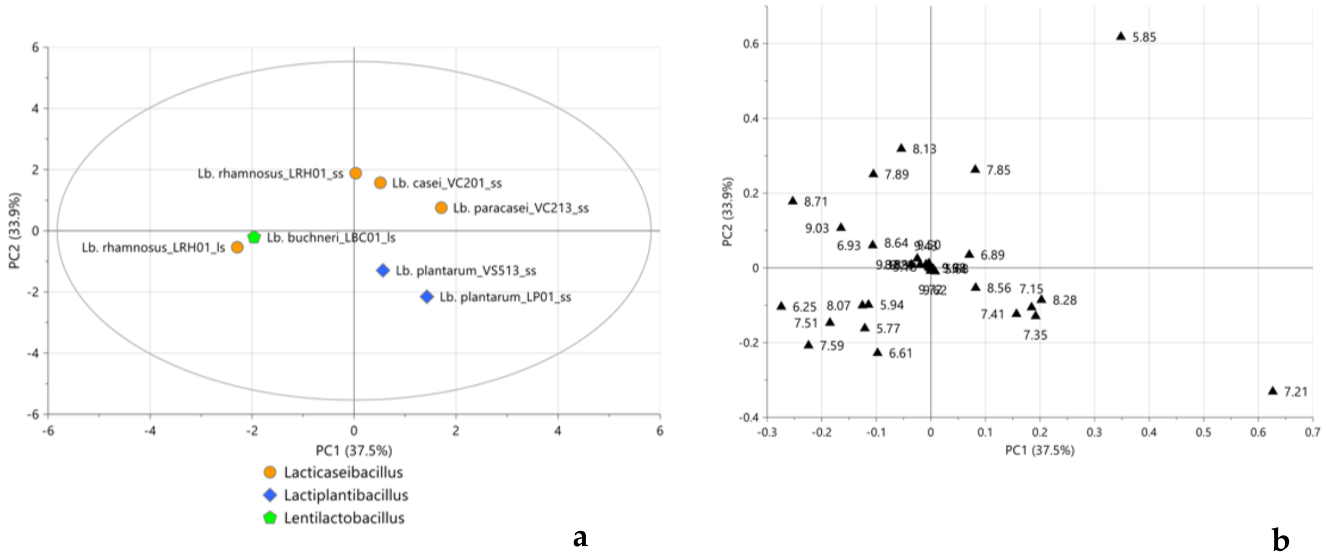

Figure S5. Score **(a)** and loading **(b)** plots of PCA performed on LAB-fermented flours considering only genera in the latest taxonomy and the aromatic region of ^{1}H NMR spectra. In **(a)**, orange circles, blue diamonds, and green pentagons represent *Lacticaseibacillus*, *Lactiplantibacillus*, and *Lentilactobacillus* samples, respectively; in **(b)** black triangles represent the loadings of the variables of X matrix (the numbers refer to the initial ppm values of the buckets). 3PCs, R^2X=89.9%, Q^2cum= 30.8%.

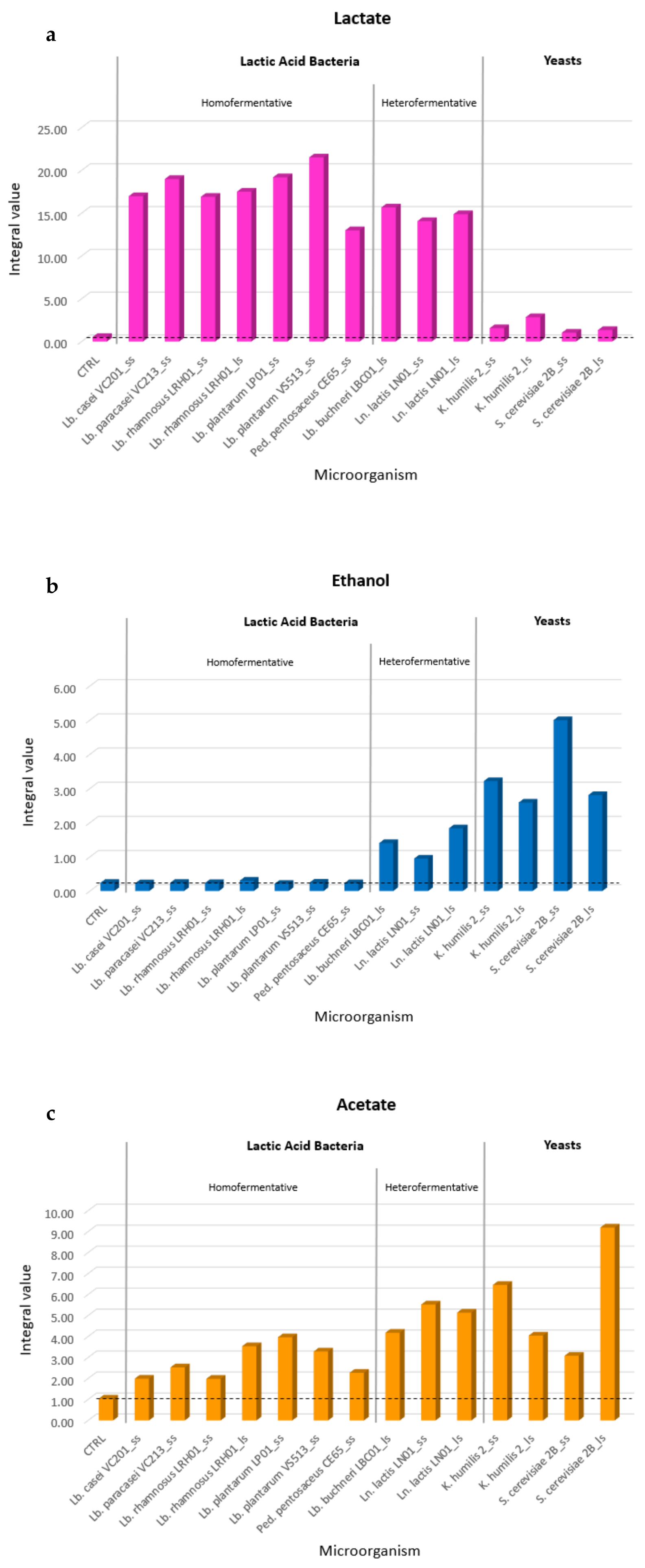
a
Lactate
Lactic Acid Bacteria
Yeasts
Homofermentative
Heterofermentative
Integral value
25.00
20.00
15.00
10.00
5.00
0.00
CTRL
Lb. casei VC201_ss
Lb. paracasei VC213_ss
Lb. rhamnosus LRH01_ss
Lb. rhamnosus LRH01_ls
Lb. plantarum LP01_ss
Lb. plantarum VS513_ss
Ped. pentosaceus CE65_ss
Lb. buchneri LBC01_ls
Ln. lactis LN01_ss
Ln. lactis LN01_ls
K. humilis 2_ss
K. humilis 2_ls
S. cerevisiae 2B_ss
S. cerevisiae 2B_ls
Microorganism
b
Ethanol
Lactic Acid Bacteria
Yeasts
Homofermentative
Heterofermentative
Integral value
6.00
5.00
4.00
3.00
2.00
1.00
0.00
CTRL
Lb. casei VC201_ss
Lb. paracasei VC213_ss
Lb. rhamnosus LRH01_ss
Lb. rhamnosus LRH01_ls
Lb. plantarum LP01_ss
Lb. plantarum VS513_ss
Ped. pentosaceus CE65_ss
Lb. buchneri LBC01_ls
Ln. lactis LN01_ss
Ln. lactis LN01_ls
K. humilis 2_ss
K. humilis 2_ls
S. cerevisiae 2B_ss
S. cerevisiae 2B_ls
Microorganism
c
Acetate
Lactic Acid Bacteria
Yeasts
Homofermentative
Heterofermentative
Integral value
10.00
9.00
8.00
7.00
6.00
5.00
4.00
3.00
2.00
1.00
0.00
CTRL
Lb. casei VC201_ss
Lb. paracasei VC213_ss
Lb. rhamnosus LRH01_ss
Lb. rhamnosus LRH01_ls
Lb. plantarum LP01_ss
Lb. plantarum VS513_ss
Ped. pentosaceus CE65_ss
Lb. buchneri LBC01_ls
Ln. lactis LN01_ss
Ln. lactis LN01_ls
K. humilis 2_ss
K. humilis 2_ls
S. cerevisiae 2B_ss
S. cerevisiae 2B_ls
Microorganism

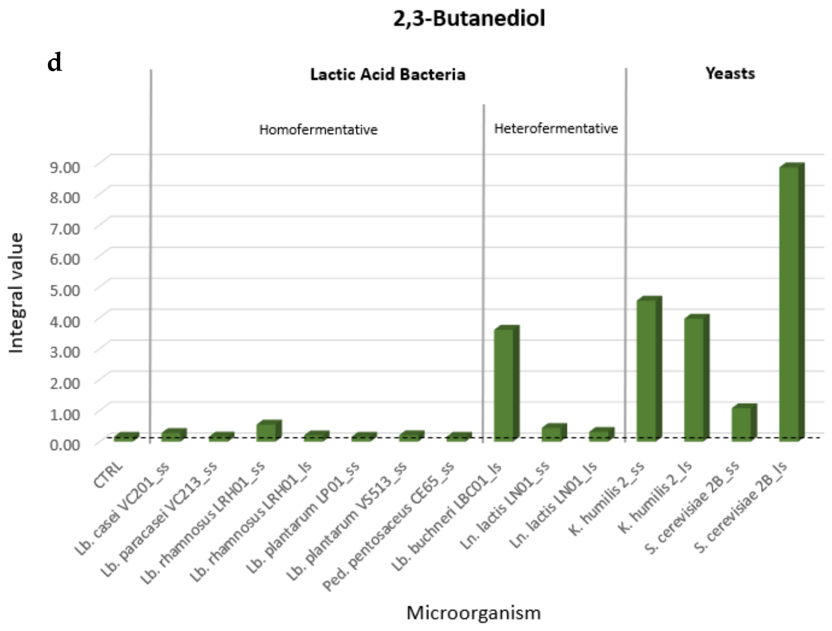

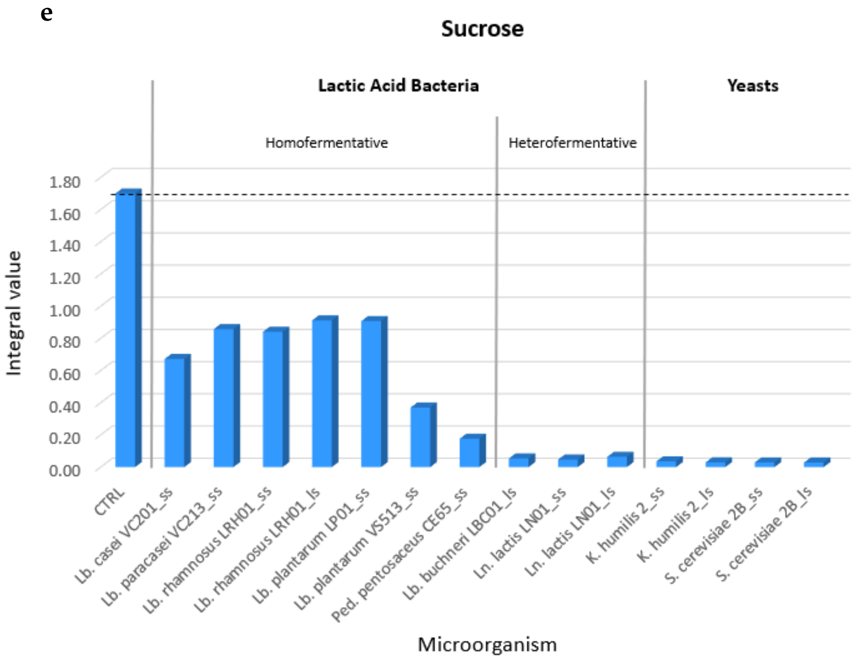

Figure S6. Quantification of the relative content (integral values normalized to the total spectral area) of fermentative metabolites: **(a)** lactate, **(b)** ethanol, **(c)** acetate, **(d)** 2,3-butanediol, and **(e)** sucrose in small- (ss) and large-scale (ls) fermented bean flours (with the exclusion of LBC01_ss, and WS01_ss samples) compared to control (CTRL). The dotted line indicates the integral value, for each metabolite, in CTRL sample.
